# Heterogeneous Immunoassay Using Channels and Droplets in a Digital Microfluidic Platform

**DOI:** 10.3390/mi10020107

**Published:** 2019-02-05

**Authors:** Yuguang Liu, Ian Papautsky

**Affiliations:** 1 Microbiome Program, Center for Individualized Medicine, Mayo Clinic, Rochester, MN 55905, USA; liu.yuguang@mayo.edu; 2 Department of Bioengineering, University of Illinois at Chicago, Chicago, IL 60607, USA

**Keywords:** digital microfluidics, enzyme-linked immunosorbent assay (ELISA)

## Abstract

This work presents a heterogeneous immunoassay using the integrated functionalities of a channel and droplets in a digital microfluidic (DMF) platform. Droplet functionality in DMF allows for the programmable manipulation of discrete sample and reagent droplets in the range of nanoliters. Pressure-driven channels become advantageous over droplets when sample must be washed, as the supernatant can be thoroughly removed in a convenient and rapid manner while the sample is immobilized. Herein, we demonstrate a magnetic bead-based, enzyme-linked immunosorbent assay (ELISA) using ~60 nL of human interleukin-6 (IL-6) sample. The wash buffer was introduced in the form of a wall-less virtual electrowetting channel by a syringe pump at the flow rate of 10 μL/min with ~100% bead retention rate. Critical parameters such as sample wash flow rate and bead retention rate were optimized for reliable assay results. A colorimetric readout was analyzed in the International Commission on Illumination (CIE) color space without the need for costly equipment. The concepts presented in this work are potentially applicable in rapid neonatal disease screening using a finger prick blood sample in a DMF platform.

## 1. Introduction

Immunoassay is a standard approach in biomedical research and clinical diagnostics for measuring target analytes that relies on antibody–antigen binding. It is one of the most common and sensitive bio-analytical techniques, and it is used in numerous applications in hospitals and clinics. For example, the diagnostics of cardiac diseases [1,2], sepsis [3], malaria [4] and traumatic brain injury [5] rely on immunoassays to detect and quantify the disease markers. Over the past few decades, the need for immunoassays has been rapidly rising due to the discovery of new biomarkers for various diseases and the development of new clinical assays to diagnose them [6].

Among the several immunoassay formats [6,7] that have been developed to meet specific clinical needs, a non-competitive heterogeneous immunoassay such as ELISA is one of the most essential types due to its higher sensitivity; this format is thus frequently used to detect critical disease markers, such as cardiac [1,8] and hepatitis markers [9]. A typical ELISA requires the immobilization of capture antibodies on a solid surface to capture the antigens of interest. Labelled detector antibodies are then added to specifically bind to the antigen, followed by a wash to remove the supernatant that contains the interfering signal from the excess labelled detector antibodies to more accurately quantify the target antigens.

Conventional immunoassay methodology relies on well plates, and is the current gold standard for immunodiagnostics, typically requiring 100 µL sample volume for each well [4,6]. However, one of its major drawbacks is the need for large sample volumes (3–10 mL blood draw), as required for signal detection and sample preparation, especially when it is necessary to detect multiple analytes [10]. Reducing the sample volume to just a few drops of a patient’s blood (<10 µL) would be beneficial, especially for pediatric and neonatal patients [11,12]. Using microfluidic devices for immunoassay has become a popular solution [13,14,15]. These devices consist of a network of microchannels fabricated in thermoplastics and elastomers (e.g., PMMA, PDMS) [16], supporting a variety of multistep immunoassay protocols. Due to the microscale dimensions of such devices, the required sample volume is reduced by ~100X compared with conventional immunoassay.

Although microfluidic devices significantly reduce the required sample volume in the immunoassay, it still is not the most desirable solution in certain circumstances. For example, in a neonatal intensive care unit (ICU), the necessity of frequent withdrawal of even a few mL of blood from neonates for disease diagnostics and monitoring can lead to severe blood loss and the need for blood transfusion [11]. This is especially the case where multiple disease markers need to be measured and monitored frequently for personalized intervention in a timely fashion. Digital microfluidics (DMF) [17,18,19] can offer an attractive alternative, and the technique is gaining attention due to its ability to handle nL to pL discrete sample droplets in a programmable fashion. Ultimately, DMF can better support low-sample-volume immunoassay applications to monitor disease markers with largely reduced blood draw volume each time.

Despite the intrinsic ability of DMF to handle nL to pL droplets, the sample volume used in a typical DMF-based immunoassay to reach the desired limit of detection (LOD) is still ~1 μL [20,21]. Further reduced volume may increase the LOD, because the signal intensity of the supernatant adds to that of the target antigens. To maintain the desired LOD in low-sample-volume heterogeneous immunoassays in DMF, thoroughly washing the sample to remove the supernatant becomes especially crucial. In a DMF-based ELISA, the sample is washed by droplet-based serial dilution (8000× fold), which adds to operational complexity [20]. Supernatant separation followed by droplet-based sample washing has been proven to be highly efficient in reducing the background signal on the DMF chip, and could be used in various immunoassay applications [22]. However, these DMF-based ELISA rely on photomultiplier tubes (PMTs)—a costly piece of equipment that may not be easily accessible in resource-limited regions—for chemiluminescent detection due to their higher sensitivity. Therefore, a protocol for convenient and effective sample washing in low-sample-volume ELISA using the DMF platform without relying on costly equipment is needed.

In this work, we combined the functionalities of continuous flow and discrete droplets [23,24] in a DMF device to demonstrate a magnetic bead-based human IL-6 ELISA, and used the International Commission on Illumination (CIE) for colorimetric image analysis to quantify the analyte [25]. IL-6 is a critical biomarker of neonatal sepsis, a bacterial infection that can manifest in the first week of a neonate’s life, and which is by far the most common cause of childhood mortality and morbidity [26,27]. To reduce the need for blood transfusions for newborns, this assay shows that only ~60 nL of sample can be enough for a single ELISA. This corresponds to a ~15X reduction in sample volume compared with a typical ELISA in DMF. With the development of microfluidic methods for extracting plasma from whole blood [28,29], it may be possible to perform a panel of DMF-based ELISA using only a fraction of the volume from a single blood draw. Others have found that under the same experimental conditions, chemiluminescent detection can quantify lower concentrations of targets [30]. To maintain the desired 10 pg/mL LOD [20] while using nL sample volume, a continuous electrowetting virtual channel [31,32] was used to wash the sample captured by the immobilized magnetic beads instead of serial dilution. The developed ELISA protocol can be easily adapted to a wide range of heterogeneous immunoassays in DMF, and shows the potential of using DMF devices for rapid diagnostics in newborns [18] using nL quantities of sample.

## 2. Methods and Materials

**Figure 1:**
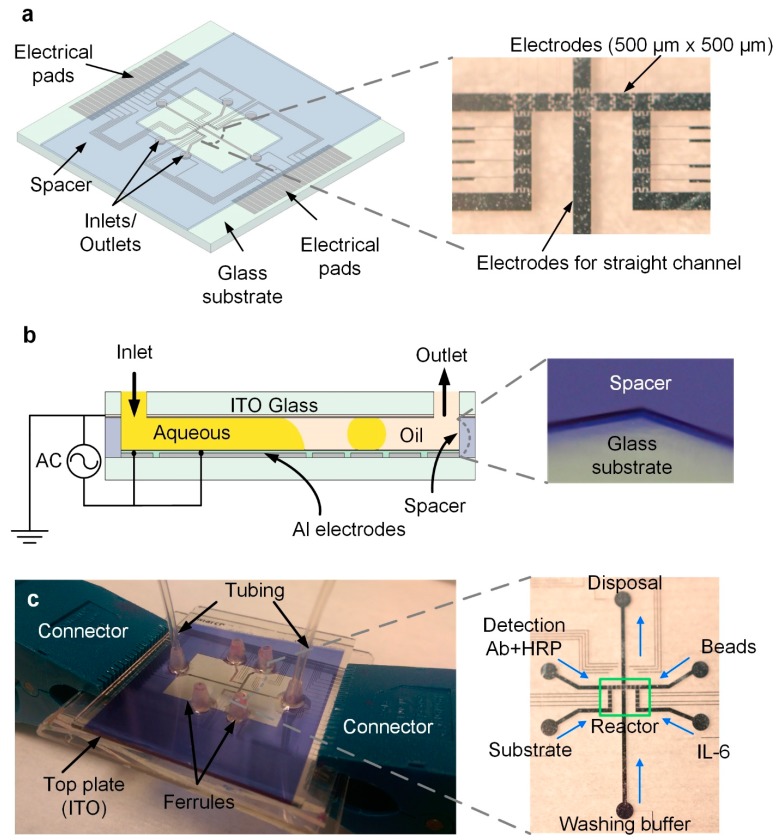
(**a**) A six inlet/outlet digital microfluidic device with 500 µm wide electrodes, allowing channel and droplet functionalities. The reaction area consists of electrode arrays for droplet manipulation. (**b**) Cross-section of the device, with Aluminum electrodes patterned on glass and insulated by a 5 µm thick Parylene C layer. A negative photoresist (PerMX 5050) was used as a spacer to define the height of the device cavity (100 μm thick). A top ITO glass substrate provided an electrical ground connection. (**c**) A completed device ready for testing. Tubes were connected to the inlet/outlet ports through the ferrules. Reagents were introduced from different ports, and droplets of samples were transported to the reactor. The washing buffer was introduced from the inlet at one end of the straight channel electrodes and flowed to the other end for disposal.

**Device fabrication.** The device in this work consisted of six inlet/outlet ports for the introduction of different reagents, as illustrated in Figure 1a. A path of 2.5 cm length, and 500 µm-wide channel electrodes, were located in the middle of the device to introduce a continuous electrowetting channel from the inlet through the outlet. An aqueous pressure-driven flow (<100 μL/min) can be formed on these channel electrodes in ambient oil in a sealed DMF device without relying on a rigid channel structure. On both sides of the straight channel electrodes, two electrode arrays (500 µm-wide) were laid out to introduce the reagents to the reactor area in the center of the device. The reactor area consisted of 15 interdigitated 500 µm-wide square electrodes, making it possible to mix and incubate the reagents. Forty electrical connection pads were placed on the two opposite ends of the device, i.e., 20 on each side. However, only 21 electrodes were laid out in this device, using ~52% of the available electrical interface.

The device fabrication followed the same procedures as those described in our earlier work [23]. The device was fabricated on a 2” × 2”, 1 mm thick glass substrate (Corning, New York, NY, USA). A 200 nm layer of aluminum was deposited to pattern the electrodes and electrical connections on the glass substrate following the standard photolithography procedure. The electrodes were insulated by a 5 µm thick layer of Parylene C (Specialty Coating Systems). A 100 µm thick spacer layer was patterned around the edges of the device using a negative photoresist MX5050 (Dupont, Wilmington, DE, USA) to define the height of the device and the area for fluid manipulation. The spacer layer also formed the physical boundaries of the circular reservoirs (1 mm) at each inlet and outlet port, preventing fluid to spread out undesirably after its being introduced into the device. A transparent conductive top plate (~200 nm ITO over glass) provided electrical ground, and contained drilled inlet and outlet ports (1mm). A 50 nm thick layer of Teflon AF 1600 (Dupont, Wilmington, DE, USA) was spin coated (1600 rpm, 60 s) on both the top and bottom glass substrates, and the substrates were heated at 160 °C for 10 min so that the surfaces became more hydrophobic (with a contact angle of ~105°) without applied potential in ambient oil (OS-30, Dow Corning, Midland, MI, USA). The ITO glass substrate was bonded to the bottom substrate using UV-curable epoxy (Dymax, Torrington, CT, USA), thus forming a cavity space sealed on all four sides except for the inlet/outlet ports. The resulting channel cross-section is schematically shown in Figure 1b. Ferrules were epoxy-bonded to the six inlet/outlet ports drilled in the top ITO glass substrate for connecting tubing to introduce reagents.

**Experimental setup.** The device was connected to an AC power source via the electrical connection pads, and was controlled by a graphic user interface (GUI) (Figure S1 in Supplementary Information). The actuation voltage of the device was 100 V, 1 kHz, and the device would last for at least 2 h without breakdown. The GUI supports the fully-automated control of electrodes in a programmed time sequence with desired intervals by setting a series of electrode activations and deactivations prior to the experiments. A completed graphic program would run throughout the entire experiment without user intervention. Such automation is much desired in the tedious task of processing a large number of volumes including on-chip sample preparation for biological and biomedical applications. However, in this work, the magnet was meticulously moved by hand with plastic tweezers, and the syringe pump was set manually when the wash step was required.

The experimental setup for the device for the ELISA experiment is shown in Figure 1c. The tubes on the syringes were connected to the ferrules to introduce reagents into the device. Different ports were used to introduce different reagents to minimize cross-contamination. On both sides of the straight channel electrodes, samples were introduced into the device first in the form of short and bended channels, and then droplets were split from the bent channel and transported to the reactor region. The washing buffer solution was introduced from the inlet and continuously flowed on the straight channel electrodes to the outlet for disposal. An external magnet (not shown) was placed on top of the device or removed, according to needs. To ensure that the beads were attracted by the magnet while still being visible to the camera, the edge of the magnet was placed on top of the beads.

**Magnetic bead functionalization.** Microbeads are often used as solid supports in microfluidic ELISA. They offer a dramatic increase in surface to volume ratio, and thus, in the number of antibodies that can be functionalized. Paramagnetic beads are especially popular because they can be easily aggregated and immobilized by an external magnet. Due to these advantages, we chose magnetic beads as a solid support to immobilize sample in the IL-6 ELISA in DMF platform. We used 1 μm N-hydroxysuccinimide (NHS) activated magnetic beads (Thermo Fisher Scientific, Waltham, MA, USA), as this is one of the simplest and most common methods for crosslinking. The NHS-activated magnetic beads will stably and efficiently conjugate with amine-containing proteins on the antibodies. In our case, the beads were functionalized with monoclonal mouse-anti-human IL-6 antibodies (Thermo Fisher Scientific, Waltham, MA, USA). The binding capacity of most proteins and antibodies on the NHS-activated beads was in the range of 21–50 μg/mg of beads [31]. To avoid wasting antibodies, we used an excessive amount of beads in our coupling, assuming the binding capacity for mouse-anti-human IL-6 antibodies on the beads to be 20 μg/mg of beads. The details of the bead functionalization follows the manufacturer’s instructions, and the coupling efficiency was measured to be ~76.7%. After the coupling, the non-reacted NHS groups were quenched using a quenching solution to prevent the direct binding of detector antibodies-HRP conjugates to the magnetic beads.

**Reagents for IL-6 immunoassay.** The ELISA was performed following the standard procedure using human IL-6 ELISA reagent kit (Life technologies, Carlsbad, CA, USA). Lyophilized recombinant human IL-6 standard was dissolved in 4% bovine serum albumin (BSA, Thermo Fisher Scientific, Waltham, MA, USA) in phosphate saline buffer (PBS, Thermo Fisher Scientific, Waltham, MA, USA, pH 7.4) filtered by 0.45 μm filter (Whatman, Maidstone, UK). The concentrations of human IL-6 standard were diluted into 1000 pg/mL, 500 pg/mL, 100 pg/mL, 10 pg/mL and 1 pg/mL respectively. Washing buffer was made from 0.04% Pluronic F127 in PBS. The detector antibodies were then functionalized with streptavidin horseradish peroxidase (streptavidin-HRP, Thermo Fisher Scientific, Waltham, MA, USA) in PBS (4:1). Blocking buffer, colorimetric TMB substrate and stop solution were included in the ELISA kit.

**Figure 2:**
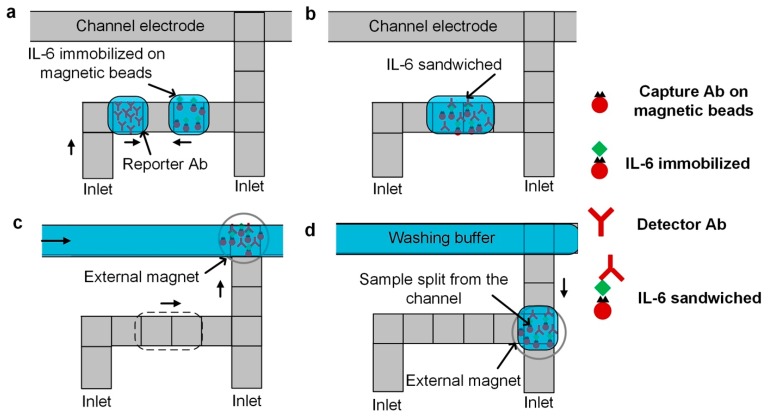
Sample washing in a heterogeneous immunoassay on a digital microfluidic platform. (**a**) Dispensing of reagents, (**b**) mixing and incubation of reagents, (**c**) introducing the droplet of immobilized sample into an electrowetting virtual channel for sample washing, and (**d**) splitting a droplet of washed sample from the electrowetting channel.

**Sample wash method in DMF-based ELISA.** The schematic of integrating a continuous channel for sample wash in our ELISA implementation is illustrated in Figure 2. Samples and reagents (e.g., bio-complexes on magnetic beads, and antibodies) are dispensed as discrete droplets (~60 nL), and these droplets are mixed to allow for a sufficient reaction to occur (Figure 2a,b). A continuous channel of washing buffer solution is introduced into the device by a syringe pump, and the reacted droplet is transported and merged into the channel while being immobilized by an external magnet (Figure 2c). The continuous flow flushed away the excess unbound bio-complexes to minimize errors in the results. After washing, a droplet of the sample is split from the channel and is ready for subsequent processes (Figure 2d). The proposed sample washing protocol can be used in a wide range of DMF-based heterogeneous immunoassays.

## 3. Results and Discussion

### 3.1. General ELISA DMF Protocol

The complete protocol for the magnetic bead-based human IL-6 ELISA in our DMF device includes seven steps. First, we dispensed a droplet of magnetic beads functionalized with capture antibodies and a droplet of human IL-6 standard. Then, the two droplets were mixed, allowing the human IL-6 molecules to be captured by the functionalized magnetic beads (0.1 s interval, 2 min). In the third step, we introduced a flow of washing buffer solution into the activated channel electrodes, merged the droplet into the channel, and immobilized the sample using an external magnet. Next, the sample-containing droplet from the channel was split and a droplet of detection antibody-HRP conjugates was dispensed and mixed with IL-6 molecules to be sandwiched. The resulting droplet was merged into the channel of washing buffer and the sample was immobilized by the magnet. A droplet of substrate solution was then dispensed and mixed with a droplet of sample split from the channel. In the final step, a droplet of stop solution was merged to terminate the reaction, and the device was imaged under an upright microscope to quantify the colorimetric readout.

**Figure 3:**
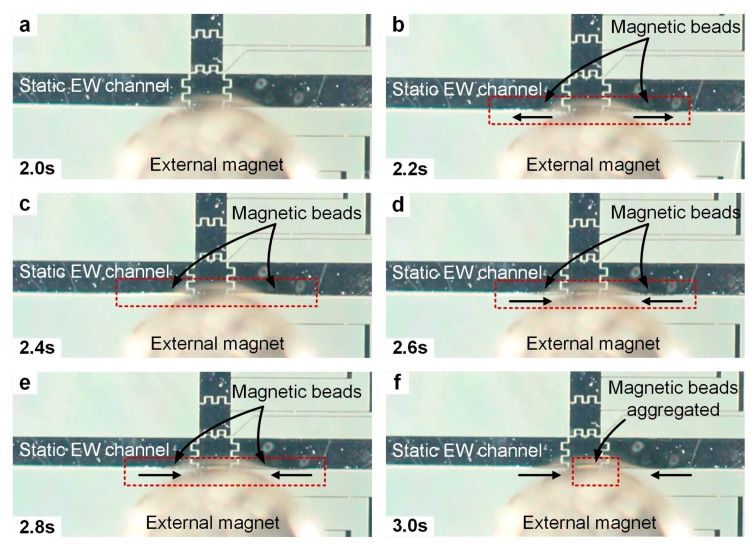
The merging of a droplet of magnetic beads into a static electrowetting channel. (**a**) A static electrowetting channel was formed. A magnet was placed on the top of the device over a droplet of magnetic beads (not shown). (**b**,**c**) The droplet of magnetic beads was merged into the channel, and the beads migrated for ~1 mm both upstream and downstream. (**d**,**e**) The beads were attracted by the magnet and migrated in the reverse directions. (**f**) The beads were aggregated on the electrode under the edge of the magnet.

To minimize bead loss during the merging of the droplet into the electrowetting channel, the flow was completely stopped before the merging (Figure 3). After the channel became static, a magnet (ND-42, 3/16”, 1 lb, ~5000 Gauss, KJ Magnetics) was placed on top of the device over a droplet of magnetic beads ready to be merged into the static channel (Figure 3a). Upon merging, the beads migrated for ~1 mm toward both upstream and downstream of the channel along the sidewall over ~1 s in the presence of the magnet (Figure 3b,c). Immediately afterwards, the migrating beads reversed their directions and were attracted toward the magnet over the next 6 s, and were aggregated on the electrode underneath the magnet (Figure 3d–f). Only after the magnetic beads were collected can the flow be resumed to wash the beads.

### 3.2. Optimization of Surfactant Concentration

One of the key challenges in current DMF-based heterogeneous immunoassay applications is the biofouling of the devices. Biofouling is caused by the non-selective adsorption of protein molecules (e.g., antibodies) onto the hydrophobic coatings of the DMF devices, preventing the further manipulation of fluids, and causing the devices to fail. The fouling of devices is very common in DMF-based biological and biomedical applications, and a low dose of Tween 20 (0.01–0.05%) is usually added to biological solutions to alleviate this problem. Nevertheless, the movement of fluid in the device is still sluggish, hindering the assay performance. To solve this problem, Pluronic F127 was added as an alternative additive [33,34] to the samples and reagents in our experiments, as Pluronic molecules form a temporary coating on the droplet interface, preventing proteins in the droplet to interact with the device surface.

The concentration of Pluronic F127 in the specific biological electrolytes used in this ELISA was tested in our device by characterizing the electrowetting contact angle. AC voltage (square wave, 1 kHz) was tested in the range of 50 to 160 V, as shown in Figure S2. These reagents were diluted in PBS according to the maximum concentration used in the ELISA, that is, 0.5 mg/mL of functionalized magnetic beads, 1000 pg/mL of IL-6, and 2000 pg/mL of detection antibody-HRP. The saturation contact angle of the reagents was ~67° at ~105 V. Under the same voltage, the droplet with Pluronic F127 was lower than droplets without the additive, which means that Pluronic F127 eases the manipulation of these biological droplets. Based on these characterization results, 0.04% of Pluronic F127 was added to the solutions, as further increases in concentration did not significantly affect the contact angle, and increased the possibility of damaging the hydrophobic coating.

### 3.3. Optimization of Magnetic Bead Retention

The use of a continuous flow is a very promising approach for sample washing in the magnetic bead-based ELISA. However, one of the potential problems is the decreased bead retention rate in the flow. Even though the beads would be immobilized by a magnet, a higher flow rate would still flush away the beads. Therefore, before demonstrating the ELISA, we optimized the process of merging a droplet of beads into the flow, the flow rate of the channel, and the process of splitting a droplet of beads from the channel to reach ~100% bead retention rate.

**Figure 4:**
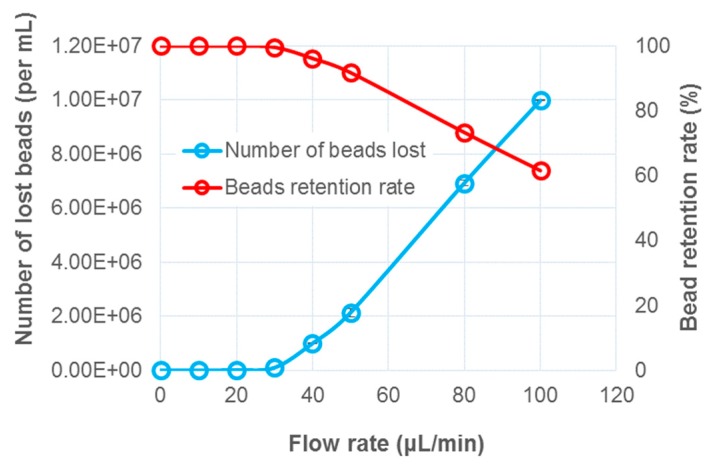
Limits of immobilizing magnetic beads using a magnet under the flow. Beads start to be flushed away with increased flow rate. ~100% bead retention rate achieved at 10 μL/min.

The flow rate of the electrowetting channel for washing the beads was tested in the range of 10–50 μL/min using a syringe pump (Figure 4). The time duration for each washing experiment lasted for 3 min. The waste wash buffer flowing from the outlet port at the end of the channel was collected and used to estimate the amount of bead loss per microliter during the washing using a hemocytometer under a microscope. When the flow rate was < 20 μL/min, ~100% bead retention was achieved. Starting from 20 μL/min, the pressure of the flow overcame the magnet pull force, and the beads were starting to escape from the magnet. Magnets with larger pull forces would aggregate the beads with greater efficiency, especially under higher flow rates; however, beads would be irreversibly aggregated, as reported by others [20]. Based on these optimization results, flowing the wash buffer at 20 μL/min would possibly lead to a faster assay; however, in this work, a flow rate of 10 μL/min was chosen for sample washing, simply as a precaution.

To ensure a high bead retention rate during the splitting of a droplet of magnetically-aggregated beads from the channel, the flow was stopped prior to any magnet movement and electrode activation and de-activation. The magnet was moved very slowly using plastic tweezers prior to any splitting, and was maintained in the same location for at least 20 s. Side electrodes were then activated to pull fluid from the channel without moving the magnet. The magnet was then moved slowly in the same manner to the farthest edge of the fluid from the channel prior to droplet splitting so that the magnet would be stationary during the splitting. No bead loss was visible when viewed using the magnified camera. Others have transported magnetic beads by moving external magnets in various experiments with minimal concern of bead loss [35,36]. However, it would be more convenient if a fixed magnet could be used [20,22]; such an approach will be pursued in future experiments.

**Figure 5:**
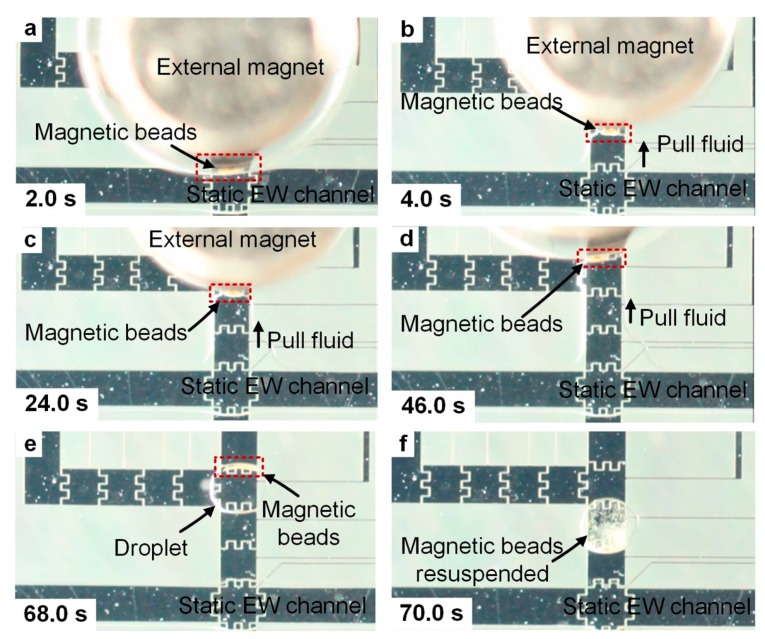
The splitting of a droplet of capture antibody functionalized magnetic beads from a static electrowetting channel. (**a**) The flow was completely stopped. A magnet was still held in place to aggregate the magnetic beads. (**b**–**d**) The side electrodes were turned on to pull the bead-containing fluid from the channel, and the magnet was slowly moved in the same direction to ensure that the beads were still aggregated. (**e**) A droplet of magnetic beads was split from the channel. (**f**) The beads were re-suspended after agitation.

Likewise, after washing, before splitting the droplet of beads from the channel, the flow should be stopped to avoid the loss of beads during this process (Figure 5a). Side electrodes were then activated to direct the fluid out of the channel, and the magnet was then slowly moved in the direction that the fluid was pulled out to ensure that the magnetic beads were still aggregated before a droplet of beads was separated from the channel (Figure 5b–d). The splitting electrode was then turned off and a droplet of magnetic beads was split from the channel. The magnet was removed after the droplet was split, but the beads were still aggregated (Figure 5e). After the droplet was agitated, the beads were then re-suspended in the droplet of washing buffer solution, and were ready to be used for the subsequent processing.

### 3.4. Colorimetric Detection of Human IL-6

Instead of using a PMT to multiply the chemiluminescent signal, in this work, we quantified the data by analyzing the colorimetric images taken by a DSLR camera connected to an upright microscope lens (20× objective). The device was transferred under an upright microscope within 5 min of the reaction being stopped. A Canon Ti3 DSLR camera body was connected to a microscope (20× objective) to capture the images. An obvious approach for processing colorimetric images is to directly convert the RGB values of the area of interest into the analyte concentration. However, the RGB intensities of the analytes did not show a discernable trend, and were difficult to correlate with the concentration. Therefore, we chose the CIE color space to code the colorimetric images for quantification [25].

In the CIE color space, each color is represented by parameter x, determining its chromaticity, and parameter y, representing its luminance in a 2-D chromaticity diagram [37,38], thereby compensating the ambient light. In this work, images were taken under the microscope within 10 min after termination of the reaction, and the images were processed by a program written in MATLAB for a three-step color space conversion, as described in detail in our previous work [25,39]. Briefly, the nonlinear RGB values obtained from the colorimetric images were first converted to linear RGB values, and the linear RGB values were then converted to tristimulus values. The chromaticity values *x* and *y* were then obtained from these tristimulus values, which can be represented in a 2-D chromaticity diagram. The IL-6 concentration should be a function of the chromaticity value x and y in the color space, and changes in the concentration shift these values.

**Figure 6:**
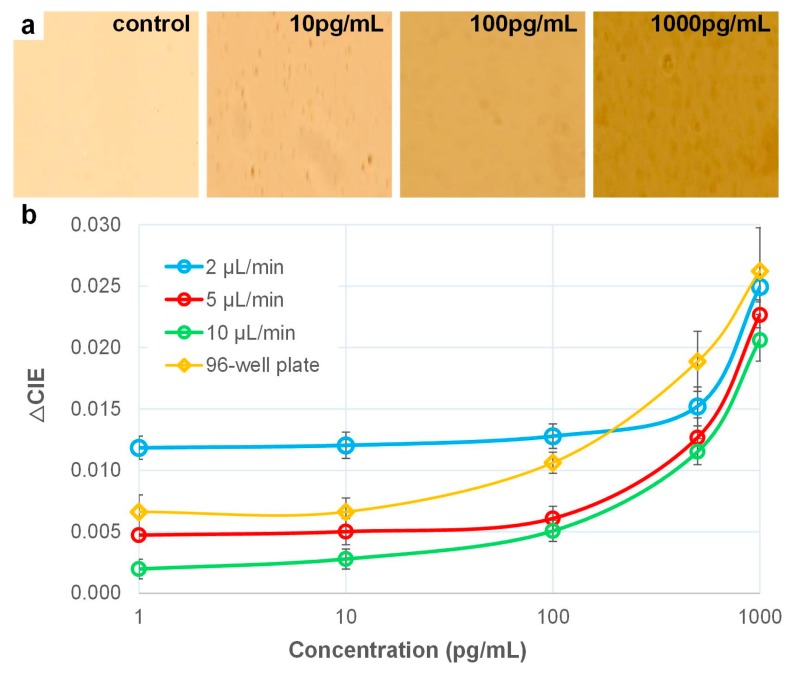
Results of on-chip and 96-well plate human IL-6 colorimetric ELISA using CIE color space analysis. (**a**) Colorimetric images of immunoassay in DMF platform at 100× magnification. Images were captured 5–10 min after the reaction was stopped. (**b**) Color signal as a function of IL-6 concentration. Differences in color are represented by the distance of shift of the mapped location in the color space relative to the mapped negative control (∆CIE).

Using this approach, the colorimetric images of the human IL-6 of different concentrations, shown in Figure 6a, were mapped onto the diagram with a set of specific value x and y. The shift toward yellower region in the diagram indicates a higher concentration of IL-6 molecules (Figure S3), and the concentration measurements are relative to each other.

It was possible to distinguish the difference in the color in all five concentrations with the naked eye looking through the microscopic objective. However, capturing images which quantify the signal strength remains a challenge. The ambient lighting conditions and camera parameters were carefully set and tuned to ensure uniform lighting. It would be more desirable to image all five samples on a single frame to ensure uniform lighting to obtain reproducible results. The images were analyzed using the CIE color space, where the difference of colors is represented by a difference in value, that is, ∆CIE. The shift toward the yellower region in the color space indicates a higher concentration of IL-6 molecules. Hence, the concentration measurements are relative to each other. The LOD for human IL-6 reached 10 pg/mL, even though the droplet volume of IL-6 was reduced to ~60 nL. As a comparison, the standard 96-well plate ELISA using the same colorimetric detection approach was performed for comparison. Both sets of results are shown in Figure 6b.

Washing the sample using a continuous flowing electrowetting channel is potentially advantageous in this DMF-based ELISA. The washing efficiency depends on the duration of the washing and the flow rate of the channel. The channel flow rate was characterized for washing efficiency in these experiments, while time duration in each washing was set to 3 min. Experiments were repeated using channels of 2 μL/min, 5 μL/min and 10 μL/min with the rest of the experimental parameters remaining unaltered (Figure 6b). The term ∆CIE refers to the distance of a mapped concentration relative to the mapped negative control in the CIE color space. At 2 μL/min, the supernatant would not be easily flushed away, especially when the beads were aggregated by the magnet. The background signal was comparatively high; therefore, the measured LOD was ~100 pg/mL, while the calculated LOD was 43.9 pg/mL. As the flow rate was increased to 5 μL/min, the washing of the sample was more efficient as more pressure was supplied to flush over the aggregated beads, lowering the calculated LOD to 21.9 pg/mL. As the flow rate was increased further, to 10 μL/min, the measured LOD was lowered to 10 pg/mL, while the calculated LOD was 18.9 pg/mL. These experimental measurements agree with the results reported by others in DMF platforms [20,21], and with data from the assay kit manufacturer that indicates a working range of 7.8–2500 pg/mL.

## 4. Conclusions

In this work, we demonstrated the integrated use of a continuous electrowetting channel and discrete droplets in a magnetic bead-based human IL-6 ELISA in a DMF device. Such a technique combines the unique advantages that a continuous channel and discrete droplet can offer in a single application. The discrete droplets allow for the use of significantly reduced sample volumes (~60 nL) in an ELISA, which is potentially attractive in clinical settings, especially for sensitive and reliable diagnostics and for the screening of neonatal diseases. For example, IL-6, IL-8 and C-reactive proteins are often targeted as a panel of biomarkers [21,40] for reliable and sensitive diagnoses of neonatal sepsis, rather than merely IL-6. The viability of using nL sample volumes for an immunoassay opens up the possibility of splitting microliters of droplets of blood samples into a number of nL of droplets for parallel immunoassays during a neonatal health screening. The use of pressure-driven continuous flow permits the effective washing of the immobilized sample in a convenient and easy-to-automate fashion, achieving 10 pg/mL of LOD by colorimetric detection in CIE color space without the need for costly detection equipment such as PMT. The demonstrated on-chip DMF protocol for the magnetic bead-based IL-6 ELISA can be extended to a wide range of heterogeneous immunoassays and other biological and biomedical applications which require the washing of immobilized samples. The integration of continuous and discrete functionalities in a programmable DMF platform opens up the possibilities of performing a wider variety of biomedical applications on a single DMF device.

## Supplementary Materials

The following are available online at http://www.mdpi.com/2072-666X/10/2/107/s1, Figure S1: GUI used to control automated electrode activation and deactivation. (a) The GUI allows for defining the electrode layout according to customized devices. Each discrete red block indicates one electrode on the DMF device. (b) Each discrete red block indicates an activated electrode, and each discrete gray block indicates a de-activated electrode. Figure S2: Electrowetting characteristics for relevant biological solutions w/o surfactant Pluronic F127. AC voltage from 50–160 V was tested. (a) PBS buffer solution (b) 0.1 mg/mL capture antibody functionalized magnetic beads suspended in PBS (c) 1000 pg/mL human IL-6 standard in PBS (d) 2000 pg/mL detection antibody-HRP conjugates in PBS. Figure S3: Colorimetric image analysis in CIE color space. A shift in the color space toward yellower region corresponds to the higher concentration of IL-6 molecules.
