# Supplementary material for: Heterogeneous Immunoassay Using Channels and Droplets in a Digital Microfluidic Platform
Source: Micromachines (Basel). 2019 Feb 5;10(2):107. doi: 10.3390/mi10020107 (PMC6412725; doi:10.3390/mi10020107)
Supplement: Supplementary file 1 [file micromachines-10-00107-s001.pdf]

Supplemental Information: Heterogeneous Immunoassay Using Channels and Droplets in a Digital Microfluidic Platform

Yuguang Liu [1] and Ian Papautsky [2,*]

[1] Microbiome Program, Center for Individualized Medicine, Mayo Clinic, Rochester, MN 55905, USA; liu.yuguang@mayo.edu

[2] Department of Bioengineering, University of Illinois at Chicago, Chicago, IL 60607, USA

[*] Correspondence: papauts@uic.edu; Tel.: +1-312-413-3800

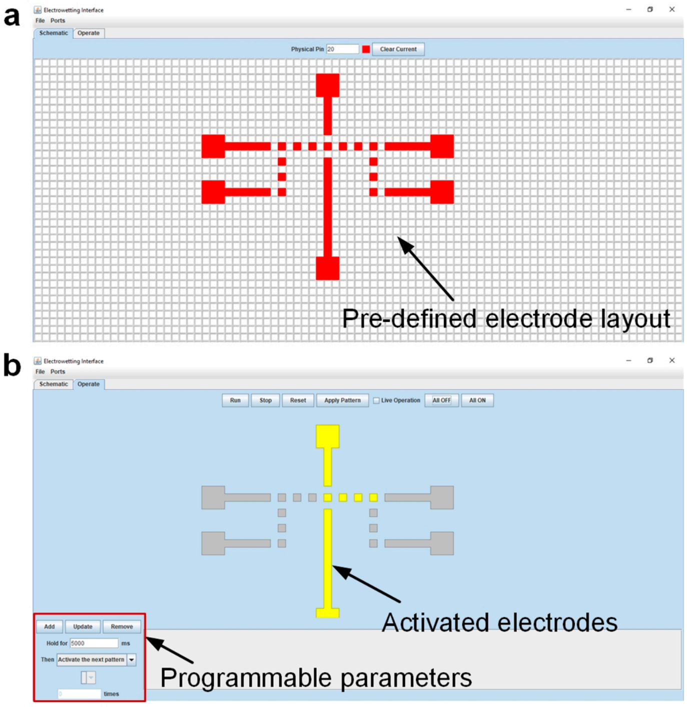

Figure S1. GUI used to control automated electrode activation and deactivation. (**a**) The GUI allows for defining the electrode layout according to customized devices. Each discrete red block indicates one electrode on the DMF device. (**b**) Each discrete red block indicates an activated electrode, and each discrete gray block indicates an de-activated electrode.

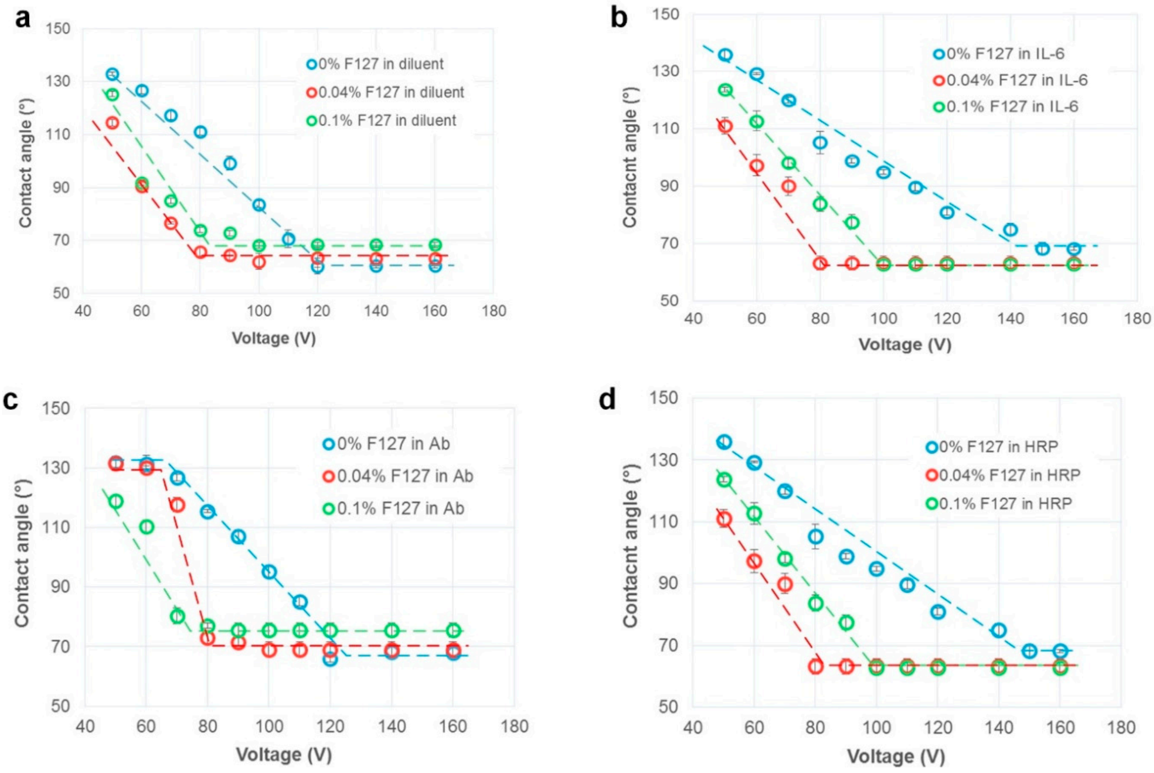

Figure S2. Electrowetting characteristics for relevant biological solutions w/o surfactant Pluoronic F127. AC voltage from 50–160 V was tested. (**a**) PBS buffer solution (**b**) 0.1 mg/mL capture antibody functionalized magnetic beads suspended in PBS (**c**) 1000 pg/mL human IL-6 standard in PBS (**d**) 2000 pg/mL detection antibody-HRP conjugates in PBS.

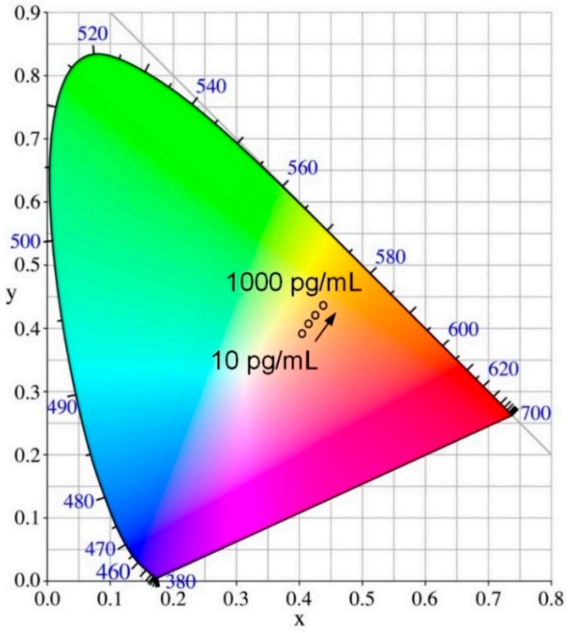

Figure S3. Colorimetric image analysis in CIE color space. A shift in the color space toward yellower region corresponds to the higher concentration of IL-6 molecules.
